# Molecular Characterization, SNP-Based Strain Profiling, and Seroprevalence of *Bacillus anthracis* in Ruminants in Jordan

**DOI:** 10.3390/microorganisms14071483

**Published:** 2026-07-07

**Authors:** Amin A. Aqel, Mohammad Abu Lubad, Hamed Alzoubi, Daniel S. Schabacker, Sara Forrester, Scott Schlueter, Mark Khemmani, Alan J. Wolfe, Tahir Yaqub, Muhammad Waqar Aziz, Mohammed Alsbou, Yasser Gaber

**Affiliations:** 1 Department of Microbiology and Immunology, Faculty of Medicine, Mutah University, Al-Karak 61710, Jordan; aminaq@mutah.edu.jo (A.A.A.); or m.abulubad@amsu.edu.jo (M.A.L.); 2 Faculty of Medicine, Aqaba Medical Sciences University, Aqaba 77110, Jordan; 3 Department of Pathology and Microbiology, Faculty of Medicine, Jordan University of Science and Technology, Irbid 22110, Jordan; hmalzoubi6@just.edu.jo; 4 Argonne National Laboratory, Lemont, IL 60439, USA; dschabacker@anl.gov (D.S.S.); sforrester@anl.gov (S.F.); sschlueter@anl.gov (S.S.); 5 Department of Microbiology and Immunology, Loyola University Chicago, Chicago, IL 60660, USA; mkhemmani@luc.edu (M.K.); awolfe@luc.edu (A.J.W.); 6 Institute of Microbiology, University of Veterinary and Animal Sciences, Lahore 54000, Pakistan; tahiryaqub@uvas.edu.pk (T.Y.); waqar.aziz@iub.edu.pk (M.W.A.); 7 Department of Microbiology, The Islamia University of Bahawalpur, Bahawalpur 63100, Pakistan; 8 Department of Pathological Sciences, College of Medicine, Ajman University, Ajman P.O. Box 346, United Arab Emirates; 9 Department of Microbiology and Immunology, Faculty of Pharmacy, Beni-Suef University, Beni-Suef 62511, Egypt; yasser.gaber@pharm.bsu.edu.eg

**Keywords:** *Bacillus anthracis*, anthrax, one health, zoonosis, molecular typing, Jordan, PCR diagnostics

## Abstract

Anthrax is an endemic and undercharacterized zoonotic disease in the Middle East, including Jordan. Between 2018 and 2020, we conducted a comprehensive investigation of 13 confirmed anthrax outbreaks in Jordan, analyzing 822 samples from animal farm environments, including carcasses, asymptomatic livestock, and abiotic environmental surfaces. All samples were tested by qPCR targeting the chromosomal marker (*ba*177) and the plasmid marker pXO1 (*pag*). Among carcass samples, 75/195 (38.5%) were *ba*177-positive, of which 81% harbored the pXO1. Of specimens from live-animals and from environmental surfaces, 218/627 (35%) were positive by qPCR, likely reflecting environmental contamination during active outbreak periods. Serological analysis using anti-protective antigen (PA) ELISA revealed a high seroprevalence of 53% (75/141) among asymptomatic animals, indicating widespread sub-clinical exposure to *B. anthracis* antigens and previously undocumented endemicity. An integrated approach combining qPCR with ELISA demonstrated that 11% of seropositive animals with paired swab testing also yielded swab samples that were positive by qPCR, suggesting environment-to-host transition. Molecular strain typing using canonical SNP (canSNP) analysis identified the rare sublineage C.USA.A1055 within lineage C.Br.A1005 across two distinct outbreaks, suggesting the environmental persistence of this endemic lineage. Overall, our findings provide the first systematic molecular and serological surveillance baseline data for Jordan, demonstrating a complex genomic persistence and subclinical exposure landscape. This study suggests the need for enhanced surveillance strategies under the One Health framework to mitigate the risk of anthrax endemicity.

## 1. Introduction

Anthrax, caused by the spore-forming *Bacillus anthracis* (*B. anthracis*) bacterium, is a formidable zoonotic threat to animal health in both natural and agricultural settings, with infections endangering food supplies and farmers’ livelihoods. Anthrax also poses a significant threat to human health, with the vast majority of human *B. anthracis* infections occurring through contact with infected animals and animal products, particularly during handling and butchering of carcasses [1,2,3]. While the potential use of *B. anthracis* spores as a biological weapon represents a biosecurity concern, direct animal-to-human transmission through contact with infected livestock and consumption of contaminated meat remains the primary epidemiological pathway [2].

*B. anthracis* spores are environmentally stable and are associated with very high fatality when untreated following inhalational exposure [3]. A major challenge to livestock health management is the widespread presence and persistence of *B. anthracis* spores in agricultural environments. Despite *B. anthracis* spores being identified in soils across the globe, including the Middle East, there is a significant diagnostic gap in distinguishing the route and causes of active infection from environmental contamination during field investigation [4,5,6,7,8,9]. *B. anthracis* can persist in isolated natural environments, including remote regions within game preserves, as well as in agricultural areas such as livestock farms [10,11]. In diagnosis, traditional bacterial culturing methods are definitive but are often precluded in field and resource-limited investigations due to high-level biosecurity requirements. Consequently, there is a need for multi-marker molecular identification, prevailing strain profiling, and sero-prevalence that can provide a comprehensive diagnostic approach in outbreak investigations. However, in endemic regions, current surveillance relies mainly on passive reporting of livestock animal mortality, which often underestimates the real scale of bacterial circulation.

Reliable molecular detection of *B. anthracis* is complicated by the presence of closely related *Bacillus* species and potential environmental contamination during outbreak investigations. Multi-marker PCR approaches targeting both chromosomal and plasmid genes can improve specificity and provide safer alternatives for field surveillance [12]. Similarly, serological assays can provide valuable information about historical exposure patterns in affected populations, complementing molecular detection for comprehensive outbreak assessment. Furthermore, single-nucleotide polymorphism (SNP) analysis of *B. anthracis* in endemic regions is lacking, limiting the understanding of the global phylogeography of *B. anthracis*.

In this study, we conducted the first systematic molecular and serological surveillance during 13 confirmed anthrax outbreaks in Jordan to establish baseline detection patterns and exposure levels. Our objectives were to: (1) evaluate dual-marker qPCR assays (chromosomal *ba*177 and plasmid *pag*) for reliable outbreak confirmation in carcass materials; (2) assess the extent of environmental *B. anthracis* contamination on affected farms; (3) investigate molecular detection patterns in apparently healthy livestock during outbreak periods; (4) characterize serological evidence of historical *B. anthracis* exposure in local populations; and (5) provide preliminary molecular typing data for Jordan isolates. This surveillance approach was designed to provide practical baseline data for outbreak investigation protocols while distinguishing between confirmed infections and environmental contamination.

## 2. Materials and Methods

### 2.1. Outbreak Definition and Site Selection

During the period between 2018 and 2020, an integrated molecular and serological surveillance project monitored confirmed anthrax outbreaks on Jordanian farms. An outbreak was defined as one or more sudden livestock mortality with clinical signs consistent with anthrax (sudden death, bloody discharges, lack of rigor mortis) as reported and investigated by Ministry of Agriculture veterinarians, with subsequent laboratory confirmation. During this period, we collected samples from 13 confirmed anthrax outbreaks across multiple Jordanian governorates. When notified of suspected anthrax cases, qualified veterinarians visited outbreak sites and collected samples according to standardized protocols. Mapping of outbreak epicenters was performed using GPS coordinates transceivers.

For each carcass, samples included blood smears, mouth swabs, nasal swabs, rectal swabs, hoof swabs, wound exudates (when present), and soil samples from beneath the carcass. From asymptomatic animals (clinically healthy herd-mates), we collected mouth, nasal, rectal, and hoof swabs, as well as serum samples for serological analysis. Environmental samples included swabs from workers’ hands and boots, fence rails, and soil from various farm locations. Samples were collected in triplicate at each site, with one representative sample from each triplicate used for subsequent analysis. After quality control procedures, a total of 822 samples were processed by qPCR, and 141 serum samples from asymptomatic animals were analyzed by ELISA.

### 2.2. Biosafety and Sample Collection Strategy

All the samples were collected by trained veterinarians with all the required personal protective equipment (PPE). To avoid the spread of spores and environmental contamination, the carcass was placed on its side on a plastic sheet, positioned away from people and other animals. The animal’s front leg was raised to expose the axillary area, and a sterile scalpel was used to create a small incision. Glass slides were used to collect capillary blood drops, with a second clean slide run at a sharp angle to create blood smears. All surfaces contacting the carcass were disinfected and sterilized. Following the initial field assessment, the owner identified asymptomatic animals from the same herd for additional sampling.

### 2.3. Live Animals and Environmental Matrix Sampling

A total of 822 biological and environmental samples were collected to check pathogen spread across the animal farm. Serum samples were collected from a subset of animals for serological analysis following standard veterinary procedures, with samples processed and stored according to established protocols. Swab samples were collected from each animal using sterile swabs. Deep nasal swabs were obtained by inserting sterile swabs into the animal’s nostril and rubbing in circular motions along the nasal walls before placing them in transport medium. Superficial nasal swabs were collected from the shallow nasal area and submerged in 5 mL Stuart’s transport medium (Hardy Diagnostics, Santa Maria, CA, USA). Oral swabs were obtained from inside the animal’s mouth to collect saliva, then submerged in 5 mL Stuart’s medium. Rectal swabs were collected from the rectal walls and placed in Stuart’s medium. Hoof swabs were obtained from hoof surfaces using the same protocol. This sampling process was repeated on selected live animals from each outbreak. Each sample was assigned a serial number and labeled appropriately.

For sampling abiotic materials in the vicinity of carcasses, 130 g of soil was collected with a sterile spoon from different locations around the carcass. Three soil samples were taken from directly underneath the carcass at 1 cm depth, where blood had oozed and stained the soil. Soil that was mixed with non-hemorrhagic fluid was also sampled. Additionally, a soil sample was taken in all four cardinal directions (north, east, south, and west) as negative controls. Each of the samples was stored in a sterile zipper bag. Swab samples and blood serum samples were stored at −20 °C. Soil samples were labeled and stored at room temperature.

**Figure 1:**
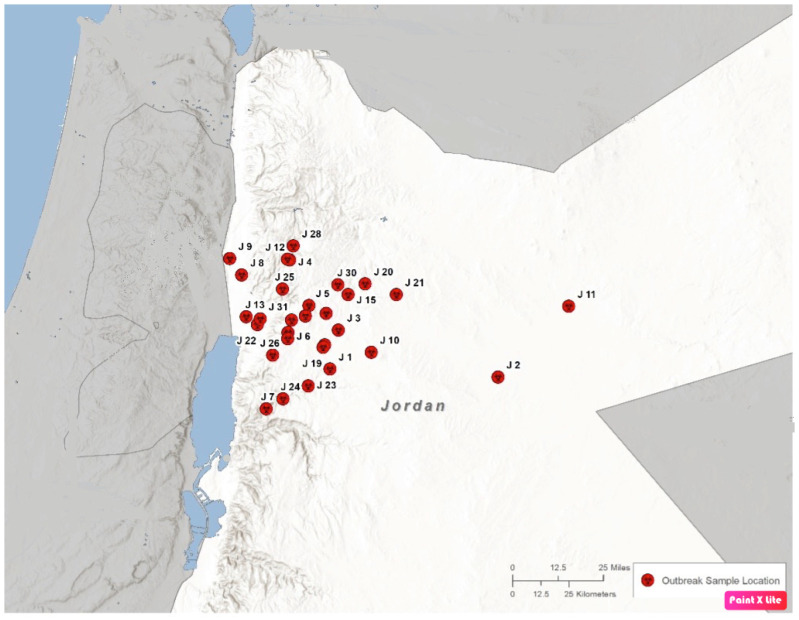
Geographical distribution of the anthrax outbreaks recorded in Jordanian locations during the current project (e.g., J23; Jordan outbreak no. 23).

GIS coordinates were recorded to map the locations of confirmed anthrax outbreaks (Figure 1). The owner determined the site of the carcass and recorded GPS coordinates using the mobile application “My GPS Coordinates”. The accuracy of GPS differed according to the location of the carcass, but all were within 2–4 m.

Samples were mainly kept and processed at Mutah University, Jordan, for preliminary identification, at Argonne National Laboratory, IL, USA, for serological analysis, and at Loyola University Chicago, Maywood, IL, USA, for qPCR and molecular identification.

### 2.4. Microscopic Examination of Anthrax Outbreaks

Microscopy was performed to determine the presence of *B. anthracis* in collected samples. For carcass blood smears, we used a Romanowsky-type rapid stain (Diff-Quik) for preliminary morphology only (e.g., visualization of large Gram-positive bacilli); Gram and Malachite Green stains were used on non-blood samples.

### 2.5. Molecular Confirmation

DNA extraction was performed according to instructions for the Qiagen DNeasy PowerSoil Kit with additional steps to remove potential Foot-and-Mouth Disease (FMD) virus RNA. Briefly, blood smears (reconstituted in 600 μL sterile water), soil (250 mg), or swab samples (400 μL transport medium with swab) were added to PowerBead tubes and gently vortexed. 60 μL of solution C1 was added to each sample, and tubes were secured horizontally in the PowerLyzer 24 homogenizer. Samples were homogenized at maximum speed for 10 min, then centrifuged at 10,000× *g* for 30 s. The supernatant was transferred to clean collection tubes. RNase A was added to a final concentration of 10 μg/mL and incubated at room temperature for 15 min to remove potential FMD viral RNA, as specified in USDA Permit 143307. Samples were then filtered and sterilized through 0.22 μm centrifuge filter tubes (Millipore, MA, USA) to remove viable bacterial cells. The remaining kit protocol was followed according to the manufacturer’s instructions. DNA was eluted using 100 μL nuclease-free water from the spin column and stored at −80 °C until analysis.

### 2.6. Quantitative PCR (qPCR)

Quantitative PCR of DNA extracted samples was performed as follows at Loyola University Chicago, Maywood, IL, USA. A total reaction volume of 10 μL contained 2 μL of sample DNA, 0.3 μL forward and reverse primers (final concentration 300 nM), and 5 μL of 2× EvaGreen supermix (Bio-Rad Laboratories, Hercules, CA, USA). Primers were directed at *ba*177 [13] and Protective Antigen *pag* [14] (Table 1). Cycling temperatures were the same for both sets of primers, 98 °C 2 min, followed by 40 cycles of 98 °C 30 s, 58 °C 15 s, and 60 °C 45 s. A standardized concentration of *B. anthracis* Sterne DNA was used to aid in the determination of sample DNA concentration. The range of detection was between 2 pg/μL–20 ng/μL of *B. anthracis* Sterne DNA. The samples were processed in duplicate. Each qPCR run included no-template (water) controls and a *B. anthracis* Sterne genomic-DNA positive control. Because an intercalating dye (EvaGreen) was used, a sample was scored positive only when the expected product amplified with a quantification cycle (Cq) at or below the Cq of the lowest reliably detected standard (2 pg/µL Sterne DNA) and produced a melt-curve peak matching the Sterne control; reactions with no amplification, later amplification, or non-specific melt profiles were scored negative. In-house cross-reactivity testing against closely related *B. cereus* group species (e.g., *B. cereus*, *B. thuringiensis*) was not performed, which we acknowledge as a limitation.

### 2.7. canSNP Analysis

Molecular strain typing was performed using canSNP analysis following the previously described methodology [15]. Probes and primer sequences were purchased from Thermo Scientific using the published probes and primers. 10 μL reactions contained 5 μL ABI Universal Master mix, 0.25 μL of premixed probe with primer (200 nM probe and 900 nM primer), and 2 μL sample template. Thermal cycling parameters were 50 °C for 2 min., 95 °C for 10 min. followed by 45–50 cycles of 95 °C for 15 s and 57 °C for 1 min.

### 2.8. Enzyme-Linked Immunosorbent Assay

Serum samples obtained from the outbreaks were assessed for the presence of antibodies specific for anthrax protective antigen (PA). Briefly, microtiter plates (Thermo Fisher Scientific, Waltham, MA, USA) were coated overnight with 0.5 μg/mL rPA (List Biological Laboratories Inc., Campbell, CA, USA) in bicarbonate coating buffer (R&D Systems, Minneapolis, MN, USA) at 4 °C. Plates were washed twice with phosphate-buffered saline (PBS) supplemented with 0.05% Tween-20 (Thermo Fisher Scientific, MA, USA) (PBST). Plates were blocked with protein-free blocking buffer (Thermo Fisher Scientific, MA, USA) and incubated for 1 h at room temperature. Plates were washed twice before the addition of triplicate test and control sera at a 1:20 dilution in blocking buffer. This was followed by 1 h incubation at room temperature. Afterward, the plates were washed three times, and anti-sheep IgG horseradish peroxidase (Novus Biologicals, Centennial, CO, USA), anti-goat IgG + HRP (Novus Biologicals, CO, USA), or anti-bovine IgG + HRP (Invitrogen, Waltham, MA, USA) were added to respective wells and incubated for 1 h at room temperature. The plates were washed three times, after which TMB substrate (Thermo Fisher Scientific, MA, USA) was added and incubated in the dark for 10 min. 50 μL of stop solution (1N sulfuric acid) was added to each well. Positive controls were obtained from species-matched vaccinated animal sera (see Table S1, Supplementary File for details). Negative controls were purchased as commercially available “normal serum” and were also species-matched. Each sample was matched by species during analysis; thus, sheep samples were compared only to sheep negative controls. The cutoff for each species was calculated as the mean of the corresponding negative control group plus three standard deviations, and this value was used as the species-specific threshold. The absorbance was read at 405 nm using the Spark multimode plate reader (Tecan, Zurich, Switzerland). The ELISA results were interpreted as binomial data (positive/negative) with the threshold set at the mean plus three SD of the negative control for the respective species to determine a measure of the antibody response. All samples were analyzed in triplicate, and although formal intra- and inter-assay coefficients of variation were not calculated, the use of triplicate measurements and species-matched controls was intended to ensure analytical consistency.

### 2.9. Statistical Analysis

All binomial proportions and their 95% confidence intervals (Table 2, Table 3 and Table 4) were calculated using the Wilson score interval. Differences in anti-PA seroprevalence across administrative districts (Table 4) were assessed using Fisher’s exact test with Monte Carlo simulation (10,000 replicates) to accommodate districts with small, expected cell counts. A two-sided *p* < 0.05 was considered statistically significant.

## 3. Results

### 3.1. Outbreaks Recorded in Jordanian Districts

In this study, we characterized 13 confirmed farm outbreaks in Jordan using molecular (qPCR) and serological (ELISA) tests. A total of 822 samples were collected from 13 confirmed outbreaks (2018–2020) in Jordan from various districts affected by anthrax. (Table S1, Supplement File).

### 3.2. Microscopy and Molecular Analysis of Anthrax Outbreaks in Jordan

**Figure 2:**
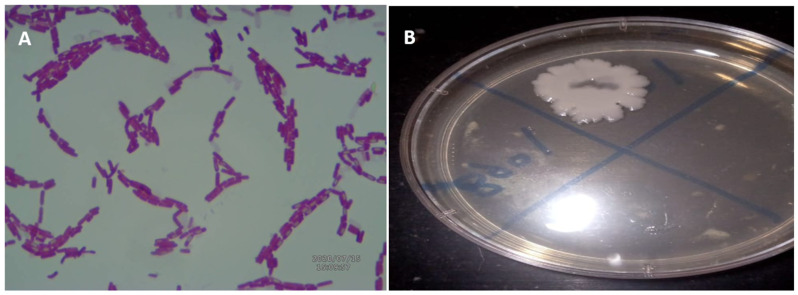
Staining and culturing for *B. anthracis*. (**A**) Positive Gram staining. (**B**) Positive growth on selective media (PLET).

Preliminary checks and analyses were conducted in Jordan at the start of the project. For microscopic analysis, Gram and Malachite Green stains were used on non-blood samples, while a Romanowsky-type rapid stain (Diff-Quik) was employed for blood smears as a morphology screen. Primary results indicated positive staining of *B. anthracis* in blood but negative results from the nose, mouth, anus, and other sites. We speculate this may be due to low spore loads in these samples. About 136 blood smears from all 13 outbreaks revealed bacilli and spores consistent with *Bacillus* spp., but capsule staining was not performed. In Gram staining, purple Gram-positive long rods with square edges were observed in long chains (Figure 2). Spore staining showed green spores inside pink vegetative cells. Microscopy identified bacilli consistent with *Bacillus* spp. in available carcass smears across all 13 outbreaks; these presumptive findings were later confirmed with qPCR.

### 3.3. qPCR Results

**Figure 3:**
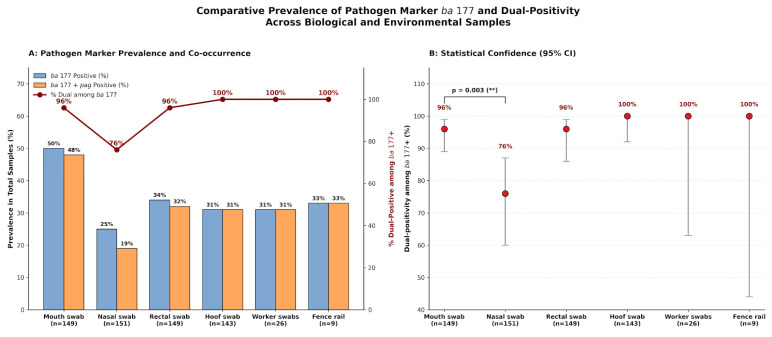
Comparative prevalence of *ba177* and *pag* markers. (**A**) The figure demonstrates the prevalence rate across biological and abiotic environmental samples. Blue bars represent the percentage of samples positive for the *ba*177 marker, while orange bars indicate dual-positivity (*ba*177+ and *pag*+). (**B**) Conditional dual-positivity, i.e., the percentage of *ba177*-positive specimens that were also *pag*-positive, shown with 95% Wilson score confidence intervals; sample sizes (n) are indicated below each category. ** *p* < 0.01.

A total of 822 specimens were screened by duplex qPCR for the chromosomal marker *ba*177 and the pXO1-encoded toxin gene *pag*. Samples from animal carcasses and positivity rates varied across sample types are presented in Table 2 and Figure 3. Due to field biosafety constraints and carcass-handling policies, blood smears were feasible in only two carcasses; confirmation, therefore, relied on multiple carcass swab types. The two carcass blood smears were used for morphology screening only; capsule staining was not performed. Diagnostic confirmation in carcass materials relied on qPCR positivity for *ba*177 and *pag*.

Mouth swabs showed 49% positivity for *ba*177, with 73% of these also carrying *pag*. Nasal swabs were positive in 39% of cases, with 82% of those dual-positive. Wound exudates and rectal swabs exhibited *ba*177 positivity in 58% and 33% of samples, respectively, with dual-positivity rates for both exceeding 80%. Hoof swabs showed 61% positivity, of which 82% were dual-positive. Soil samples collected from beneath carcasses showed limited detection, with 9% *ba*177 positivity (3 samples); all three positives also carried *pag*. Overall, dual-positivity among *ba*177-positive carcass specimens was 81% (95% CI 70.7–89.4).

### 3.4. qPCR Detection in Live-Asymptomatic Animals and Environmental Specimens

A total of 627 specimens were collected from asymptomatic animals and environmental sources on affected farms. Of these, 218 (35%) were positive for the chromosomal marker *ba*177. Within this group, 204 specimens (33% of all samples) were also positive for the pXO1-associated *pag* marker, while 14 (2%) carried *ba*177 alone. No specimen was positive for *pag* in the absence of *ba*177. Detection rates varied across sample types (Table 3). Among asymptomatic animals, mouth swabs showed *ba*177 positivity in 75/149 samples (50%), rectal swabs in 50/149 samples (34%), hoof swabs in 45/143 samples (31%), and nasal swabs in 37/151 samples (25%). Dual-positivity rates among *ba*177-positive samples were as follows: mouth swabs 96% (72/75), rectal swabs 96% (48/50), hoof swabs 100% (45/45), and nasal swabs 76% (28/37). Environmental specimens included worker swabs from hands and boots, which showed *ba*177 positivity in 8/26 samples (31%), and fence rail swabs, which were positive in 3/9 samples (33%). All positive environmental samples were also positive for *pag*. The overall total dual-positivity among *ba*177-positive specimens from both live animals and environmental sources was 94% (204/218) with a 95% confidence interval of 90–96%.

### 3.5. Serological Analysis of Asymptomatic Animals

**Figure 4:**
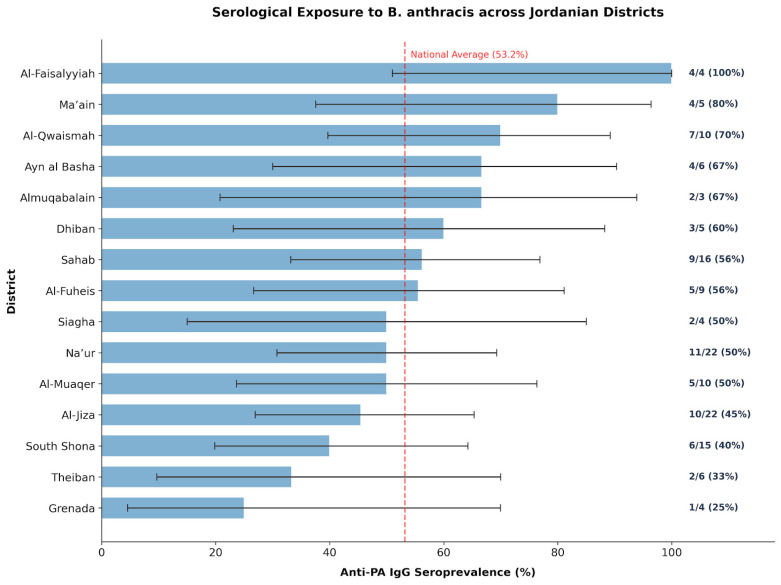
**Serological exposure to subclinical *****B. anthracis***** across Jordanian districts. **Horizontal bars represent the percentage of animals testing positive for antibodies against Protective Antigen (PA) via IgG ELISA. Data were collected from 141 apparently healthy animals in the vicinity of confirmed outbreaks (2018–2020). District-level prevalence is shown with 95% confidence intervals (Wilson Score interval), illustrating regional heterogeneity in exposure risk. The vertical dashed line (red) indicates the national mean seroprevalence (53.2%). Labels on the right-hand margin provide the specific sampling ratio (number of positive animals over total tested) and the calculated percentage for each administrative district. High seropositivity in districts such as Al-Faisalyyiah and Ma’ain indicates significant historical exposure and pathogen endemicity in these locales.

Live-asymptomatic animals’ blood specimens were analyzed for an immune response to *B. anthracis* by ELISA detection of antibodies specific to protective antigen (PA). Asymptomatic blood from all 13 confirmed outbreaks in Jordan was analyzed. Table 4 and Figure 4 display ELISA-PA data in Jordan; a total of 141 live, asymptomatic animals were tested. Overall, 75 animals tested positive for PA, representing 53% of the total samples.

We analyzed results from 93 animals that had both PCR and ELISA testing performed. PCR testing was conducted on swab samples (mouth, nose, rectum, hoof), while ELISA tested serum for antibodies against PA. Of the 54 animals that were ELISA-positive, 6 also had positive PCR results from swab samples, while 48 were PCR-negative. Among the 39 animals that were ELISA-negative, 3 had positive PCR results from swabs, and 36 were negative for both tests. In total, 9 animals had positive PCR results from swab samples, and 84 were PCR-negative.

### 3.6. canSNP Analysis

**Figure 5:**
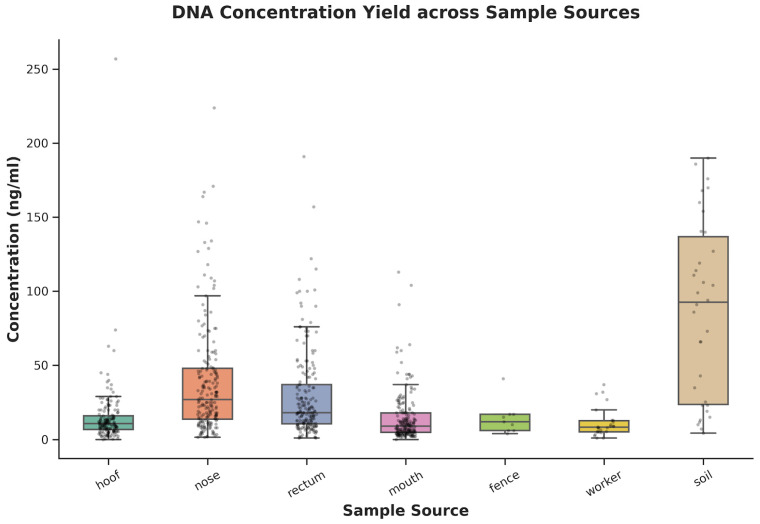
**Quantitative assessment of sampled matrices.** DNA concentration yields (ng/μL) across primary sample sources. Data are presented as box plots (median and interquartile range) overlaid with individual data points to illustrate distribution density and extraction variance.

**Figure 6:**
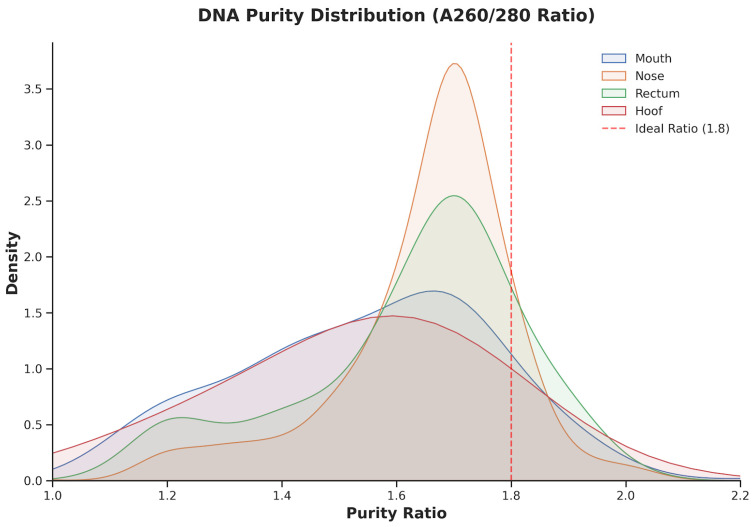
**Qualitative assessment of sampled matrices.** Kernel Density Estimate (KDE) of DNA purity (A260/A280 ratio) for major biological sites. The dashed vertical line at 1.8 represents the ideal purity threshold for downstream molecular applications.

**Figure 7:**
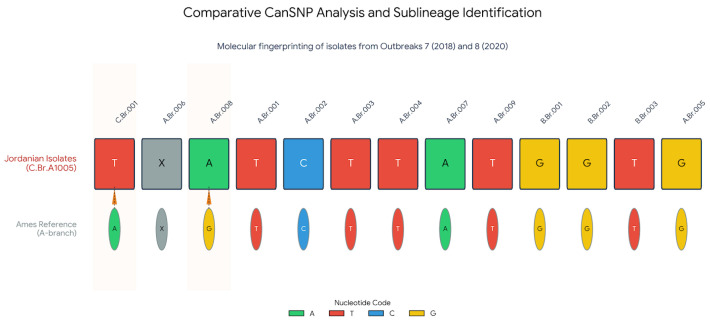
Comparative canSNP profiling and sublineage identification. Detailed molecular fingerprint of the Jordanian isolates compared against the standard *B. anthracis* Ames reference strain. Colored boxes represent the 13 canonical SNP loci used in the Keim/Pearson typing scheme. Orange indicators and background shading highlight the diagnostic polymorphisms (e.g., at C.Br.001 and A.Br.008) that define the rare C.Br.A1005 lineage. Nucleotide codes are colored according to international bioinformatics standards (A: Green, T: Red, C: Blue, G: Yellow).

**Figure 8:**
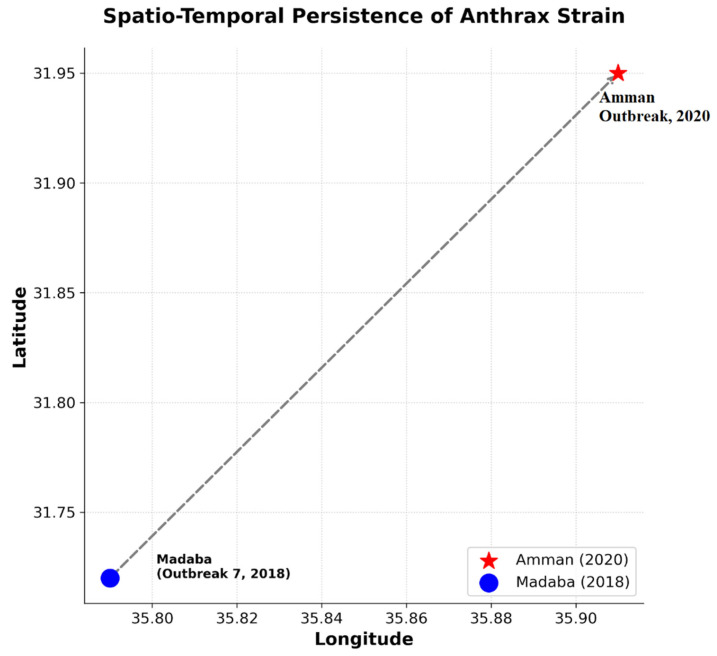
**Spatio-temporal mapping and hypothesized genetic linkage of endemic *****B. anthracis***** strains. **The arc connecting the 2018 Madaba (Outbreak 7) and 2020 Amman (Outbreak 8) isolates represents an identical canSNP profile match (sublineage C.USA.A1055). Genetically, the pairwise distance between these two isolates across all evaluated canonical SNP loci is zero (0 SNPs), establishing a direct clonal relationship.

A total of 53 samples that had tested positive by qPCR were checked for DNA quantification and purity (Figure 5 and Figure 6) before canSNP genotyping. However, many of these extracts had limited remaining volume or degraded quality due to the inactivation procedures required for export, including treatment for FMD virus and sterility validation. Thus, among these 53 samples, only 2 gave conclusive results. A sample was collected from rectal swabs of sheep associated with outbreaks in Madaba (Outbreak 7, 9 September 2018) and Amman (Outbreak 8, 13 July 2020). Both isolates had identical canSNP sequences (TXATCTTATGGTG) and were identified as belonging to the sublineage C.USA.A1055 (Table 5). Calculated pairwise genetic distance between the Madaba and Amman isolates revealed zero chromosomal alterations (0 SNP differences) across the evaluated loci. Detailed comparison of SNP allele patterns with the Ames reference strain is shown in Figure 7. As illustrated by the linkage arc in Figure 8, this absolute genetic conservation across a 22-month interval provides strong molecular evidence of stable local persistence. 

## 4. Discussion

The primary aim of this study was to provide baseline evidence on anthrax outbreaks in Jordan using molecular and serological tools. Rather than proving transmission pathways, our work sought to document the presence of *B. anthracis* markers in carcasses, apparently healthy livestock, and environmental samples collected during 13 confirmed outbreaks. While molecular detection in live animals was limited, serological analysis revealed widespread historical exposure to *B. anthracis* antigens in the study population. These observations add to the limited surveillance data available from the Levant region and contribute to baseline information on anthrax occurrence in endemic areas.

This study combined qPCR, ELISA, and limited canSNP analysis to characterize samples collected between 2018 and 2020. Together, these approaches provided complementary information on the occurrence of anthrax markers and antibodies across different sample types. The qPCR results revealed detection patterns among carcass, environmental, and live-animal samples, confirming the utility of direct molecular screening in outbreak investigations. Our dual-marker assay (*ba*177 + *pag*) effectively identified *B. anthracis* DNA in carcass materials, corroborating outbreak etiology across multiple farms. The diagnostic design aligns with the multi-marker evaluation of Ochai et al. [12], who demonstrated that combining chromosomal markers (e.g., Ba-1) with plasmid-encoded virulence genes (*pag*) yields superior diagnostic sensitivity and specificity. Their findings support the validity of our two-marker approach, which prioritizes biosafety and diagnostic precision over redundant marker inclusion. In contrast to culture-based confirmation, which is often restricted in Jordan, direct qPCR detection provides a safe, rapid, and internationally recognized method for confirming anthrax outbreaks [12].

The geographic distribution of outbreaks, illustrated in Figure 1, showed multiple reports near Amman, particularly in Na’ur, Al-Jiza, and Sahab. These districts yielded the largest number of samples and outbreaks, likely reflecting higher livestock densities and closer human–animal contact, patterns consistent with other studies in endemic settings [16,17,18]. Additional activity was noted in the South Shona and Al-Fuheis districts, while isolated events occurred in the Grenada and Siagha districts. These observations highlight the importance of geographically targeted surveillance, but statistical evidence for clustering could not be established from the available data.

Analysis of sample types revealed expected patterns. Carcass-derived materials showed high qPCR positivity (38.5%), consistent with the bacterial loads typically associated with acute infections. In contrast, lower positivity rates in samples from live animals (35%) and environmental materials likely reflect environmental contamination or lower spore concentrations. Among asymptomatic animals, positive swab qPCR results must be distinguished from active infections. For example, mouth and rectal swabs may acquire spores from contaminated feed, water, or soil during outbreak periods. Environmental samples, including fence rails and worker materials, also yielded positives, demonstrating the capacity for spores to persist in farm environments, as previously reported [19].

Again, the relatively high positivity in mouth and rectal swabs from asymptomatic animals should be interpreted with caution. In many cases, these results may reflect external contamination rather than true infection. Mouths and rectums could be contaminated with spores from feed, soil, or water, particularly in grazing animals. In addition, spores may be transferred indirectly through dust, water runoff, or contact with contaminated carcasses. Our inclusion of abiotic surfaces, such as soil and equipment, was intended to capture these early transfer events. The aim was not to map full farm-to-farm transmission but to characterize how carcasses may serve as the initial source of contamination for animals and surrounding materials.

An important observation in this study was the detection of samples positive for the chromosomal *ba*177 marker but negative for the pXO1-associated *pag* gene. This discrepancy may result from plasmid loss, differential degradation of chromosomal and plasmid DNA under environmental conditions, or low target concentrations near the assay detection limit. Consequently, detection of the *ba*177 marker alone provides less definitive evidence for the presence of fully virulent *B. anthracis* strains than the concurrent detection of both chromosomal and plasmid targets. These findings underscore the importance of multi-target molecular assays for improving diagnostic confidence in anthrax surveillance.

Furthermore, while the detection of *B. anthracis* DNA on mucosal surfaces of live animals and within environmental matrices aligns with the regional classification of endemicity, our cross-sectional study design does not permit direct modeling of real-time transmission dynamics or definitive host-to-host transitions. The presence of genomic DNA fragments via qPCR does not automatically confirm bacterial viability, active host colonization, or shedding. Consequently, these findings should be conservatively interpreted as strong molecular indicators of wide-scale environmental exposure and potential environmental reservoirs rather than direct evidence of active transmission chains.

The serological results (Table 4) further support widespread historical exposure to *B. anthracis* across affected districts. Overall, 53% of asymptomatic animals were seropositive for antibodies against protective antigen, with particularly high rates in districts Al-Faisalyyiah (100%), Ma’ain (80%), and Al-Qwaismah (70%). Lower proportions were recorded in districts Grenada (25%) and Theiban (33%). When qPCR and ELISA results were compared, approximately 11% of ELISA-positive animals were also qPCR-positive, indicating limited concurrent detection. The majority of ELISA-positive but PCR-negative animals likely represent prior exposure, consistent with the persistence of antibody responses after exposure events [20]. The small number of ELISA-negative but PCR-positive animals may reflect recent exposure before the development of detectable antibody levels, consistent with known timelines for immune activation in anthrax [21,22]. Henceforth, further research is needed to understand the dynamics of *B. anthracis* exposure and immune responses in endemic settings, particularly the relationship between environmental contamination and serological evidence of historical exposure.

The seropositivity observed in asymptomatic animals suggests prior exposure to *B. anthracis*. Notably, none of the animals had been vaccinated before the outbreaks, which aligns with common practice in Jordan. This rules out vaccination as the source of the immune response. However, without follow-up data, we cannot determine whether these animals successfully cleared the exposure. Our findings are consistent with recent mathematical modeling work by Triska and Zevika [23], who showed how *B. anthracis* spores and infected carcasses can drive recurrent outbreaks in herbivorous animals, even in the presence of vaccination programs [23,24].

canSNP genotyping yielded interpretable results for two DNA extracts out of 53 tested (Table 5). Both originated from sheep rectal swabs collected during separate outbreaks—Madaba in September 2018 and Amman in July 2020—and displayed the same concatenated allele pattern (TXATCTTATGGTG; allele states reported as a 13-site concatemer; see Methods). The recurrence of identical genotypes across temporally separated outbreaks is summarized in Figure 8, supporting the hypothesis of environmental persistence. Under the Van Ert canonical scheme, this pattern maps to sublineage C.USA.A1055 within lineage C.Br.A1005 [15].

These assignments should be regarded as provisional. Genotyping was performed on inactivated field extracts rather than DNA from pure isolates; co-extracted DNA from other *B. cereus* group organisms and degradation from inactivation steps could bias SNP detection. In addition, canonical SNPs provide broad lineage placement and are not designed for local outbreak attribution. A major limitation of the present surveillance study was the low amplification and genotyping success rate, as interpretable canSNP profiles were obtained from only 2 of 53 analyzed samples (3.8%). This limited success is likely attributable to the use of superficial mucosal swabs collected from asymptomatic animals under field conditions, which are expected to contain substantially lower quantities of *B. anthracis* DNA than diagnostic specimens such as blood or tissue samples obtained from clinically affected or deceased animals. Consequently, the genetic dataset generated in this study was restricted to only two successfully typed isolates collected over a two-year period. Given this limited sample size, robust phylogenetic reconstruction and comprehensive evolutionary analyses could not be reliably performed. Although both genotyped samples were assigned to the C.USA.A1055 sublineage, suggesting potential persistence of a locally circulating strain within the study region, this observation should be interpreted cautiously and considered preliminary. Prior reports from Jordan have documented other lineages (e.g., A.Br.008/009) [25]; if confirmed by culture-based typing (e.g., MLVA or WGS-SNP) alongside pXO2 assessment, the present calls would expand the catalog of lineages circulating in the country.

An important design limitation in the current study is that sampling was outbreak-based rather than population-based: asymptomatic animals and environmental samples were collected from herds on farms with laboratory-confirmed anthrax, selected for outbreak investigation rather than by probability sampling. Consequently, the 53% seroprevalence and molecular detection rates characterize exposure within outbreak-affected farms and must not be extrapolated to Jordanian livestock as a whole; population-representative estimates would require a randomized, nationwide sampling frame.

Future surveillance efforts should include standardized species documentation to enable more detailed epidemiological analysis. From a One Health perspective, the integration of animal, environmental, and molecular data in this study demonstrates how cross-sector collaboration can enhance outbreak detection and response to zoonotic threats. Such coordinated surveillance is essential for preventing spillovers to humans and ensuring sustainable livestock health management in endemic regions.

## 5. Conclusions

This study provides the first systematic molecular and serological surveillance data for anthrax outbreaks in Jordan, demonstrating the effectiveness of dual-marker qPCR for outbreak confirmation while establishing baseline exposure patterns in the region. Among 822 samples from 13 confirmed outbreaks (2018–2020), molecular detection effectively confirmed *B. anthracis* in carcass materials (38.5% positivity), supporting the use of *ba*177/*pag* dual-marker assays for field diagnostics. Surface molecular detection across asymptomatic live animals was recorded in 35% of specimens; however, this positivity reflects environmental contamination and surface exposure rather than active host infection. In contrast, serological analysis revealed widespread historical exposure (53% seroprevalence), indicating previous contact with *B. anthracis* antigens across the study population. These findings support enhanced surveillance strategies that combine molecular outbreak confirmation with serological monitoring for exposure assessment. The integration of animal, environmental, and molecular data demonstrates the value of coordinated One Health approaches for anthrax surveillance in endemic regions. This baseline information will support evidence-based control strategies and improved preparedness for future outbreak investigations in Jordan and the broader Middle East region.

## Figures and Tables

**Table 1:** Primers used for *B. anthracis* detection.

Target Gene	Primers	Specificity	Reference
Chromosomal marker (*ba*177)	F: TTGGATCAGCGTTTCTGAATTCAGC	*B. anthracis* chromosomal marker	[13]
	R: TCCCCATATCGCTCAATTCCATCTA		
Virulence plasmid pXO1 (*pag*)	F: CAGAATCAAGTTCCCAGGGG	Protective antigen gene (*pag*) on plasmid pXO1	[14]
	R: TCGGATAAGCTGCCACAAGG		

**Table 2:** Detection of *B. anthracis* chromosomal (*ba*177) and plasmid (*pag*, pXO1) markers in animal carcass specimens collected during 13 confirmed anthrax outbreaks in Jordan, 2018–2020.

Sample Type	Tested (*n*)	*ba*177 Positive *n* (%)	*ba*177 + *pag* Positive *n* (%)	*ba*177 Positive/*pag* Negative *n* (%)	% Dual-Positive Among *ba*177 (95% CI)
Blood	2	2 (100)	2 (100)	0 (0)	100 (34–100)
Mouth swab	45	22 (49)	16 (36)	6 (13)	73 (50–89)
Nasal swab	44	17 (39)	14 (32)	3 (7)	82 (57–96)
Wound exudate	12	7 (58)	6 (50)	1 (8)	86 (42–99)
Rectal swab	40	13 (33)	11 (28)	2 (5)	85 (55–98)
Hoof swab	18	11 (61)	9 (50)	2 (11)	82 (48–98)
Soil under the carcass	34	3 (9)	3 (9)	0 (0)	100 (29–100)
Total	195	75 (38)	61 (31)	14 (7)	81 (71–89)

**Table 3:** Detection of *B. anthracis* chromosomal (*ba*177) and plasmid (*pag*, pXO1) markers in live-asymptomatic animals or environmental specimens collected during 13 confirmed anthrax outbreaks in Jordan, 2018–2020.

Sample Type	Tested (*n*)	*ba*177 Positive *n* (%)	*ba*177 + *pag* Positive *n* (%)	*ba*177 Positive/*pag* Negative *n* (%)	% Dual-Positive Among *ba*177 (95% CI)
Mouth swab	149	75 (50)	72 (48)	3 (2)	96 (89–99)
Nasal swab	151	37 (25)	28 (19)	9 (6)	76 (60–87)
Rectal swab	149	50 (34)	48 (32)	2 (1)	96 (86–99)
Hoof swab	143	45 (31)	45 (31)	0 (0)	100 (92–100)
Worker swabs (hands/boots)	26	8 (31)	8 (31)	0 (0)	100 (63–100)
Fence rail	9	3 (33)	3 (33)	0 (0)	100 (44–100)
Total	627	218 (35)	204 (33)	14 (2)	94 (90–96)

**Table 4:** ELISA results of live-asymptomatic animals located in the neighborhood of recorded dead animals in the anthrax outbreaks.

District	Outbreak Number	Total Tested	Positive Anti-PA IgG *n* (%)	95% Confidence Interval
Al-Faisalyyiah	18	4	4 (100)	(51.0–100.0%)
Al-Fuheis	4, 12	9	5 (56)	(26.7–81.1%)
Al-Jiza	1, 10, 17, 19	22	10 (45)	(26.9–65.3%)
Al-Muaqer	2, 3	10	5 (50)	(23.7–76.3%)
Al-Qwaismah	11, 14	10	7 (70)	(39.7–89.2%)
Almuqabalain	25	3	2 (67)	(20.8–93.9%)
Ayn al Basha	28	6	4 (67)	(30.0–90.3%)
Dhiban	7	5	3 (60)	(23.1–88.2%)
Grenada	6	4	1 (25)	(1.3–69.7%)
Ma’ain	26	5	4 (80)	(37.6–96.4%)
Na’ur	5, 16, 23, 27,29	22	11 (50)	(30.7–69.3%)
Sahab	15, 20, 21, 30	16	9 (56)	(33.2–76.9%)
Siagha	22	4	2 (50)	(15.0–85.0%)
South Shona	8, 9, 13, 31	15	6 (40)	(19.8–64.3%)
Theiban	24	6	2 (33)	(9.7–70.0%)
Total	-	141	75 (53)	(44.9–61.3%)
Fisher’s Exact Test				*p* = 0.043

Asymptomatic animals were sampled, and a total of 141 samples passed the quality control criteria to proceed to ELISA testing.

**Table 5:** canSNP identification of two anthrax samples from Jordanian outbreaks.

Sample ID	Outbreak No.	Location	Date	Sample Type	Animal	canSNP Sequence	Identified Sublineage	Lineage
7D1R3Y18	7	Madaba	9 September 2018	rectum	Sheep	TXATCTTATGGTG	C.USA.A1055	C.Br.A1005
8D2R1Y20	8	Amman	13 July 2020	rectum	Sheep	TXATCTTATGGTG	C.USA.A1055	C.Br.A1005

## Data Availability

The datasets generated and/or analyzed during the current study (including sample metadata, qPCR Ct values, and ELISA serological data) are available from the corresponding author, Prof. Mohammed Alsbou (m.alsbou@ajman.ac.ae), upon reasonable request.

## Supplementary Materials

The following supporting information can be downloaded at: https://www.mdpi.com/article/10.3390/microorganisms14071483/s1, ESI-file1-Samples-PCR_vs2.xlsx, which contains metadata for 822 samples (2018–2020), including sample ID, outbreak details, sample type/source, and basic DNA lab data. Table S1, Supplementary_Table_ELISA, which provides details of the species-matched positive control sera used in the ELISA assay.
