# Supplementary material for: Molecular Characterization, SNP-Based Strain Profiling, and Seroprevalence of Bacillus anthracis in Ruminants in Jordan
Source: Microorganisms. 2026 Jul 7;14(7):1483. doi: 10.3390/microorganisms14071483 (PMC13414010; doi:10.3390/microorganisms14071483)
Supplement: Supplementary file 1 [file microorganisms-14-01483-s001.zip › microorganisms-4353468-supplementary.pdf]

Supplementary Table S1. Positive Control Sera Used for Anti-PA ELISA

Sera were obtained from vaccinated cattle and sheep and used as species-matched positive controls for ELISA assay validation and cutoff determination.

Sr. No.	Lab I.D.	Detail	Animal
1	VCS1	Vaccinated cow serum 1	Cow
2	VCS2	Vaccinated cow serum 2	Cow
3	VCS3	Vaccinated cow serum 3	Cow
4	VSS1	Vaccinated sheep serum 1	Sheep
5	VSS2	Vaccinated sheep serum 2	Sheep
6	VSS3	Vaccinated sheep serum 3	Sheep
7	VSS4	Vaccinated sheep serum 4	Sheep
8	VSS5	Vaccinated sheep serum 5	Sheep
9	VSS6	Vaccinated sheep serum 6	Sheep
10	VSS7	Vaccinated sheep serum 7	Sheep
11	VSS8	Vaccinated sheep serum 8	Sheep
12	VSS9	Vaccinated sheep serum 9	Sheep
13	VSS10	Vaccinated sheep serum 10	Sheep
